# Recent Occurrence, Diversity, and Candidate Vaccine Virus Selection for Pandemic H5N1: Alert Is in the Air

**DOI:** 10.3390/vaccines12091044

**Published:** 2024-09-12

**Authors:** Yordanka Medina-Armenteros, Daniela Cajado-Carvalho, Ricardo das Neves Oliveira, Milena Apetito Akamatsu, Paulo Lee Ho

**Affiliations:** Centro BioIndustrial, Instituto Butantan and Fundação Butantan, São Paulo 05503-900, SP, Brazil; yordanka.armenteros@fundacaobutantan.org.br (Y.M.-A.); daniela.carvalho@fundacaobutantan.org.br (D.C.-C.); ricardo.oliveira@fundacaobutantan.org.br (R.d.N.O.)

**Keywords:** HPAI H5N1 virus, H5 hemagglutinin, pandemic H5N1 epizootic, H5N1 pandemic preparedness, A (H5N1) candidate vaccine virus, A (H5) non-A (H5N1) candidate vaccine virus

## Abstract

The prevalence of the highly pathogenic avian influenza virus H5N1 in wild birds that migrate all over the world has resulted in the dissemination of this virus across Asia, Europe, Africa, North and South America, the Arctic continent, and Antarctica. So far, H5N1 clade 2.3.4.4.b has reached an almost global distribution, with the exception of Australia and New Zealand for autochthonous cases. H5N1 clade 2.3.4.4.b, derived from the broad-host-range A/Goose/Guangdong/1/96 (H5N1) lineage, has evolved, adapted, and spread to species other than birds, with potential mammal-to-mammal transmission. Many public health agencies consider H5N1 influenza a real pandemic threat. In this sense, we analyzed H5N1 hemagglutinin sequences from recent outbreaks in animals, clinical samples, antigenic prototypes of candidate vaccine viruses, and licensed human vaccines for H5N1 with the aim of shedding light on the development of an H5N1 vaccine suitable for a pandemic response, should one occur in the near future.

## 1. Introduction

Humanity was struck by the pandemic influenza viruses H1N1 in 1918, H2N2 in 1957–1958, H3N2 in 1968, and H1N1 (H1N1pdm09 virus) in 2009, resulting in millions of deaths, enormous economic losses, and lessons (to be) learned [1]. In the most serious influenza pandemic, H1N1 in 1918, it is estimated that a third of the world’s population, about 500 million people, were infected and at least 50 million died worldwide. The only countermeasures taken to control and treat the influenza infection were non-pharmaceutical interventions [2], with a limited effectiveness. Fortunately, this scenario is very different from what we experience today. Antibiotics, antivirals, and, particularly, vaccines are new countermeasures that can be used to mitigate and prevent influenza as well as associated infections based on pharmaceutical interventions [3]. The movement of the population to different regions of the planet—even to the most difficult places to reach—and the speed at which we can travel, facilitated by technological advances, are aggravating factors that were not present in 1918 and that could allow for the faster global spread of dangerous viruses. The deadliest flu pandemic also showed specific outcomes. Although the 65+ age group experiences high mortality (70–90%) due to seasonal flu infections [4], the 1918 H1N1 flu pandemic had a unique feature: adults—including those between 20 and 40 years of age—had a higher mortality rate [2]. This was similar to what was observed during the 2009 influenza pandemic when it was estimated that 80% of the deaths related to the (H1N1)pdm09 virus occurred in people under 65 years of age. In the United States (USA), it took about seven months to produce a large quantity of the monovalent vaccine against the (H1N1)pdm09 virus [4], which may have had a negative impact on the mortality in the first year of the pandemic. Therefore, influenza preparedness must be planned so as to reduce the time required to produce a pandemic vaccine, as well as to target and cover all age groups of the population. 

Another important aspect that was common to at least three out of the four influenza pandemics is that the viruses responsible for the infection had genes of avian origin [2,5,6]. Moreover, the virus causing the flu pandemic, H1N1pdm 2009, had a unique combination of genes not previously identified in humans or animals [4]. Thus, it is very important to conduct surveillance on the influenza viruses circulating in animals and humans, to perform a risk assessment of human infections, and to determine the preparedness in case of an emerging pandemic strain. This might be critical for the recent highly pathogenic avian influenza (HPAI) H5N1 virus, which has been circulating since the end of 2021 [7]. This ongoing epizootic event has the potential to become a flu pandemic and represents a public health threat. The development of candidate vaccine viruses (CVVs) and suitable potency-testing reagents that best match the circulating viruses are major aspects of pandemic preparedness, since influenza vaccination is considered the first and most important step in protecting against influenza viruses. In the present work, we reviewed some epidemiological aspects of the HPAI H5N1 viruses circulating as part of the ongoing epizootic, the clades associated with the recent lethal human cases, the licensed human H5N1 vaccines that could be used in the case of an H5N1 pandemic, the antigenic prototype of the CVVs available, and the existence of potency-testing reagents. Finally, possible CVVs were indicated against the H5N1 clades 2.3.4.4b, 2.3.2.1a, and 2.3.2.1c, which were responsible for the last reported lethal human cases.

## 2. H5N1 Influenza Virus

### 2.1. Genome and Antigenicity

The H5N1 avian influenza virus (AIV) belongs to the influenza virus A genus in the Orthomyxoviridae family. Its genome is made up of eight single-stranded negative-sense RNA segments that encode for at least eleven distinct polypeptides: three surface proteins [hemagglutinin (HA), neuraminidase (NA), and matrix 2 (M2)], three polymerases [polymerase basic (PB) 1, PB2, and polymerase acidic (PA)], PB1-F2, nucleoprotein (NP), matrix 1 (M1), non-structural protein 1 (NS1), and the nuclear export protein (NEP). The virus envelope contains two main glycoproteins, HA and NA, and based on antigenic characteristics, sixteen HA types (H1–H16) and nine NA types (N1–N9) have been distinguished. All of them have been isolated from wild birds, the natural reservoir of the influenza A virus [8].

The segmented genome of the influenza virus gives the advantage of exchanging gene segments between viruses (“antigenic shift”) when more than one virus genotype infects a single host cell. The associated influenza genes can reassort in co-infected cells, resulting in the emergence of antigenically novel viral strains and/or subtypes to which the human population is naive. Moreover, influenza viruses, like other RNA viruses, undergo rapid changes driven by the inherent mutation rate of the viral polymerase, resulting in antigenic changes (“antigenic drift”) in all the genome segments of the virus; however, this is more evident within the genes that encode exposed virulence factors that are antibody targets, such as HA and NA. Antigenic drift is generally responsible for annual influenza epidemics in humans, whereas antigenic shift is more associated with the emergence of pandemic strains [9]. Thus, H5 gene mutations enable the virus to escape the immune system and may increase its pathogenicity [10]. Therefore, it is essential to understand the evolution of these changes and the emergence of new variants, especially concerning the HA antigen.

### 2.2. AIV Pathotypes

AIVs are classified as low- or high-pathogenicity avian influenza viruses based on clinical signs in birds [11]. Two aspects involving HA are considered key virulence factors: (i) the differential HA binding affinity to α2–3 and α2–6 sialic acid receptors, and (ii) the presence of a polybasic cleavage site (PBCS). The first one is discussed in Section 3.3 (H5N1 spillover to non-human mammals). PBCSs have been so extensively found in H5N1 that the presence of a PBCS often dictates the use of the term and association to HPAI viruses. This classification is restricted to the H5 and H7 subtypes [12], and viruses are often classified as low- or high-pathogenicity avian influenza viruses based on the absence or presence of this PBCS (LPAI or HPAI, respectively) [11].

HA processing by proteolytic enzymes in the host is essential for virus infection. The cleavage of the HA0 precursor occurs in a loop that usually contains a single arginine and, rarely, a single lysine residue. Low-pathogenicity viruses possess this monobasic motif at this cleavage site [13] (cleaved by tissue-restricted trypsin-like enzymes) [12], whereas the HA of high-pathogenicity viruses contains a polybasic motif with the consensus sequence R-X-R/K-R [13] (cleaved by ubiquitous furin-like enzymes) [12]. The distribution of proteases—involved in HA processing—and the extent of their proteolytic activity are essential factors that determine the pathogenicity, spread, and tropism of the influenza virus. The trypsin-like proteases that cleave the LPAI virus are present only in a limited number of cells or tissues, so these viruses normally cause localized infections in, for example, the respiratory or intestinal tract of birds, with mild or asymptomatic infections. In contrast, furin-like proteases that activate HPAI viruses are ubiquitously expressed, causing systemic and severe infections in poultry with mortality rates of up to 100% [14,15].

Among avian influenza, viruses carrying H5 or H7 HA and presenting a PBCS are associated with a high-pathogenicity phenotype [16]. However, some H7 HPAI viruses do not have a PBCS. This has been reported in field cases for H7N1 [17] and H7N3 [18] and under experimental conditions (in chicken embryo cells) for H7N3 [19], H7N7 [20], and H5N2 [21]. The cleavability of these non-polybasic sites might be increased by the insertion of the amino acids close to the cleavage site, by non-homologous recombination [20,21]. It is believed that the insertion increases the surface exposure of the cleavage site, and thus, its accessibility to proteases [22].

## 3. Epizootic HPAI H5N1

### 3.1. Evolution and Spread of HPAI H5N1 Viruses

Since 2003, HPAI H5N1 viruses have globally disseminated in large waves, increasing the number of outbreaks affecting birds (wild and domestic). For instance, it was reported that more than 400 million domestic poultry in 63 countries died because of H5N1 from 2003 to March 2012, caused by either an infection with an H5N1 strain or animal culling in affected areas [23]. Over the last 20 years, non-human mammals have also been infected, with recent and unusual mass mortality events, while human infections have been sporadic and restricted to bird contacts. Before 2003, HPAI H5N1 virus isolates were also reported: the first reports were in chickens in Scotland in 1959 from a small farm; in turkeys in England in 1991, with about eight thousand poultry involved; and in chickens in Hong Kong in 1997, involving about three million poultry [11]. In 1997, the virus detected in Hong Kong jumped to humans, with 18 cases (six fatal) reported. This was the first known case of human infection with H5N1 [24]. It is believed that a virus circulating in domestic geese in Southern China (Guangdong Province) in 1996 was introduced to Hong Kong poultry markets in 1997, leading to the ancestral A/Goose/Guangdong/1/96 (Gs/GD) HPAI H5N1 virus lineage [25]. A Gs/GD descendant was responsible for the outbreaks in China and across Asia in 2003, and since then, it has evolved and spread throughout several continents, resulting in the currently circulating H5N1 strains [24]. The above-mentioned HPAI viruses, reported before 1996, were unrelated to this relevant lineage, and they were classified as belonging to the Eurasian-origin non-goose/non-Guangdong (EA_nonGs/GD) clade [26].

Between 1997 and 2001, the evolution of Gs/GD viruses was characterized by a rapid expansion in genotypic diversity in China [27]. In 2002, the Z genotype emerged and spread to other Southeast Asian countries, including Southern China, Cambodia, Indonesia, Thailand, and Vietnam. This genotype caused widespread outbreaks in chickens and ducks during 2003 and 2004 [28]. The Z genotype underwent changes in the neuraminidase, giving rise to a very similar genotype called Z+, which was identified from human infections in Hong Kong in early 2003. The H5N1 virus isolated from a 33-year-old man (A/Hong Kong/212/03) and his 8-year-old son (A/Hong Kong/213/03) were almost identical in all the gene segments and closely related to the Z and Z+ genotypes. This was the first detection of an H5N1 virus in humans since 1997 [29].

The infection of a broad range of hosts with Gs/GD-like viruses led to the evolution of distinct genetic lineages. Groups of viruses within the lineage were classified by following a unified nomenclature system and virus clade for H5N1 [30], based on the phylogenetic characterization and sequence homology of the HA gene. Ten distinct clades (clades 0–9) of H5 influenza A were identified, with many of these clades containing further higher-order subclades [31,32].

H5N1 spread out of Southeast Asia toward the African continent in 2005. This virus was defined as clade 2.2 and was first associated with a large die-off of wild birds in Qinghai Lake in China [33,34]. Next, it was detected in Russia, Kazakhstan, parts of Europe, Egypt, several Western African countries, India, and Bangladesh. This provided convincing evidence that wild migratory birds play a role in the epidemiology and spread of this virus and disease [35,36]. According to Harfoot and Webby, “the next substantial change in Gs/GD epidemiology came with the emergence of two distinct lineages of virus in South-East Asia: 2.3.2 and 2.3.4” [36]. The genetic drift led the 2.3.2 lineage to diverge into further groups. In mid-2009, the clade 2.3.2.1a moved westward, with detections in Russia and then Europe [37], whereas in 2015, 2.3.2.1c viruses were detected in Russia and Africa, following the previously predicted airways [36].

The unexpected emergence of clade 2.3.4.4 reinforced the idea that Gs/GD evolution is driven by both shift and drift mechanisms. The Gs/GD viruses 2.3.4.4 started to globally dominate outbreaks from 2014 onwards [38], entering for the first time into the USA and Canada in late 2014 [39,40]. The H5 HAs from this clade are predominantly, but not only, of the H5N1 subtype; however, they have been associated with different neuraminidase types [41]. The HA clade 2.3.4.4 of H5 viruses was further divided into eight subclades, named 2.3.4.4a to 2.3.4.4h. The variant 2.3.4.4b emerged in October 2020 as a particularly fit virus, spreading faster and farther than any of its predecessors [42,43]. At the beginning of 2020, H5N8 viruses bearing HA clade 2.3.4.4b infected domestic poultry and wild birds, leading to the loss of over 33 million domestic birds in Europe, Africa, and Asia [44,45,46].

H5N8 2.3.4.4b was also detected in eight farm workers who participated in a response operation to contain an avian influenza H5N8 outbreak detected in a poultry farm in Astrakhan Oblast in the Russian Federation. These were the first reported detections of avian influenza A (H5N8) in humans, and the patients were asymptomatic for several weeks. In 2020, H5N8 viruses were also detected in poultry or wild birds in Bulgaria, the Czech Republic, Egypt, Germany, Hungary, Iraq, Japan, Kazakhstan, the Netherlands, Poland, Romania, and the United Kingdom [47]. Moreover, these H5N8 viruses reassorted with other AIVs and originated H5N1, H5N2, H5N3, H5N4, H5N5, and H5N6 viruses to enter the Netherlands [44,48,49]. Interestingly, H5N1 viruses have disseminated worldwide since they emerged in the Netherlands in October 2020 [7,50,51]. From July 2021 to June 2024, only H5N1 human infections by clades 2.3.4.4b, 2.3.2.1a, and 2.3.2.1c have been reported. While the 2.3.2.1c and 2.3.2.1a clades have exhibited a restricted circulation in the Asiatic region, 2.3.4.4b has circulated almost all over the world, with human cases being reported in 11 countries throughout Europe, Asia, and North and South America [52,53]. More features referring to the dissemination, mammalian adaptation, and human infections of Gs/GD H5N1 in the ongoing epizootic are discussed later in Section 3.

### 3.2. The Ongoing Epizootic H5N1 in the Americas

Outbreaks of HPAI were reported by the end of 2021, mainly in North America and Europe. These outbreaks occurred in both domestic and wild birds, with the H5N1 subtype being predominantly identified [7]. The H5N1 virus has reached the global scale, affecting countries in Asia, the Middle East, Africa, Europe, and the Americas. This virus has decimated large poultry farms due to infection or the containment measures adopted by public health agencies. In addition, wild birds, many of which are at risk of extinction, are being infected and killed by the virus. Some issues of the current epidemic call for particular attention: (i) this is the first time that H5N1 has been reported in South America, and (ii) an unusual persistence of the virus in wild birds was observed in the summer months [54,55].

HPAI H5 has not been reported in America since June 2015 [56]. Through migratory routes, the H5N1 virus made its way to North America. The first detections of the HPAI clade 2.3.4.4b viruses were in wild and domestic birds in November 2021 in Newfoundland, Canada, and they later disseminated to the USA and Mexico [50]. In January 2022, clade 2.3.4.4b H5N1 viruses were detected in multiple wild birds in North and South Carolina (USA). Genetic analyses demonstrated that all the virus segments were of Eurasian origin (99.7–99.8% similar) and had a high identity to the 2021 HPAI H5N1 detected in Newfoundland [50]. This implies that the virus was carried across the Atlantic. A phylogenetic analysis of bird genome sequences revealed that the virus in North America had a high identity to the virus circulating in Northwestern Europe in Spring 2021, and all of them were related to the 2.3.4.4b clade that has been circulating since 2020 [57].

After the first reported outbreak in Newfoundland, the virus was identified a year later from samples collected in October 2022 in Colombia (A/wild duck/Colombia/Choco/3501/2022) [58]. Thus, from late 2022 to 2024 and ongoing, H5N1 viruses are being reported in South America [59]. Our BLAST analysis of the amino acid sequence of H5 GenBank WDE94949.1 (A/Pelican/CHL/227087-1/2022)—a representative sequence of twelve H5 sequences from isolates collected from wild birds in Chile in December 2022—revealed that this H5 virus shared a >99% identity with the H5 sequences of isolates that have been circulating in the Americas since January 2022. The two sequences analyzed were GenBank UWI70278.1 (A/common eiders/Maine/W22-481A/2022, a representative H5 sequence from the analysis of 70 isolates circulating in North America from January to December 2022) and the Venezuelan isolate GenBank WAH70677.1 (A/Pelecanus occidentalis/Venezuela/Pel4S4/2022) (see reference [60]). Moreover, H5 Genbank WDE94949.1, from a Chilean bird isolate, was identical to H5 GISAID EPI2510183’s amino acid sequence, which was obtained from the Chilean human isolate GISAID EPI_ISL_17468386 (A/Chile/25945/2023) collected almost four months later [26,58].

Another remarkable feature of the ongoing epizootic in the Americas is the report of the first natural infection of H5N1 in a ruminant species. The virus was detected in ten neonatal goats displaying neurological symptoms and mortality. They had been housed in a backyard poultry establishment in Minnesota, USA [53]. In addition, since March 2024, outbreaks of avian influenza H5N1 in dairy cattle have been reported across twelve states of the USA. This is the first time that AIV H5N1 bovine has been reported. Additionally, three human cases—one in Texas (March 2024) and two in Michigan (May 2024)—were detected in dairy farm workers after they had come into contact with infected cows. The genetic analysis revealed that the viruses belonged to the Eurasian Gs/GD lineage clade 2.3.4.4b circulating in wild birds, poultry, and mammals in the USA. No neurological signs were observed, and the cows typically recovered within 2 weeks with little to no mortality [61].

On 15 May 2023, the Brazilian Ministry of Agriculture and Livestock (MAPA) notified the World Organization for Animal Health (WOAH) of the first detection of HPAI H5N1 in wild birds in the State of Espírito Santo [62]. Up until 15 March 2024, nine detections of H5N1 infections in wild birds had been reported in Brazil [53]. Two of the three most important migratory flyways in the Americas pass through Brazil, the Central Americas, and the Atlantic Americas, with Brazil being the only country that has two of the seven migratory flyways for wild bird populations in the world [63]. Therefore, epidemiological surveillance for the purpose of monitoring the global introduction and circulation of HPAI is of crucial importance, as wild birds are virus-competent reservoirs and do not recognize geopolitical boundaries. Their migration serves to spread the virus across countries and continents [33,35].

### 3.3. The H5N1 Spillover to Non-Human Mammals

HA is responsible for the entry of influenza viruses into the cell. It presents a globular head domain delimited by conserved Cys-58 and Cys-290 (H5 numbering), linked to a stalk domain and arranged in a homotrimer. The globular domain induces immunodominant neutralizing antibodies and is responsible for binding to the cellular receptor: sialic acid linked to galactose in glycoproteins and ganglioside in the host cell membrane. Many avian influenza viruses preferentially bind to sialic acid residues connected to galactose by α2–3 linkages, while human influenza viruses bind by α2–6 linkages [64]. Human cells of the upper respiratory tract have α2–6 linkages and lack α2–3, which explains why avian influenza poorly infects the epithelial cells of the upper respiratory tract. The distribution of these two types of sialyl oligosaccharides varies among avian species. The Galliformes (e.g., chickens, turkeys, and quails), Columbiformes (e.g., pigeons and doves), Gruiformes, Pelecaniformes, Gaviiformes, and Ciconiiformes orders express both types of receptors. Passeriformes and Anseriformes (e.g., ducks, geese, and swans) predominantly express α2–3 sialic acid [65]. The upper respiratory airways of pigs, an important susceptible host, present a mixture of these sialic acid linkages, so they can be infected with viruses presenting tropism to α2–3 or α2–6 linkages. The co-infection of a single cell in pigs with avian and human viruses enables the exchange of segments, resulting in the emergence of new strains or subtypes favoring antigenic shift [66]. Nevertheless, Gs/GD viruses are not known to replicate well in pigs, with a limited number of detections of HPAI H5N1 infections in pigs. The genomes of highly pathogenic H5N1 viruses were reported in Indonesian pigs during the 2005–2007 period. In the 2016–2017 season, serosurveillance in France revealed the seroconversion of one pig through the hemagglutination inhibition assay to a 2.3.4.4.b-clade virus [67]. During the ongoing epidemic, H5N1 infections in pigs have been recorded only in Italy [68].

Over more than two decades, the H5N1 HPAI viruses (Gs/GD) have evolved, circulated, and infected multiple hosts, including about 33 mammal species [52,53,68]. Most of the initial reports were related to one or several animals infected with H5N1. However, three recent outbreaks have been highlighted because of the occurrence of mass mortality events. These occurred in seals (USA, July 2022), minks (Spain, October 2022), and sea lions (Peru, January–February 2023).

In the summer of 2022, an unusual mortality event was observed in harbor and gray seals in New England (USA), which coincided with an outbreak of the HPAI H5N1 virus (related to the 2.3.4.4b clade) in wild birds in the region [69]. Some considerations in this event indicate a likely mammal-to-mammal transmission: although infected wild birds were the sources of contamination, it was unlikely that multiple seals were infected through predation or scavenging, since birds are not a typical food source for these species [68]. However, the authors concluded that the data did not support seal-to-seal transmission as a primary route of infection. On the other hand, the sequencing of samples from infected seals showed the amino acid changes E627K and D701N in the PB2 polymerase protein [69]. These mutations have been previously associated with mammalian adaptation [70].

The second unusual mortality event occurred on a mink farm, with more than 50,000 space-confined animals in Northwest Spain in October 2022. Space confinement—analogous to that found in poultry farming—can increase the transmission of these viruses. The viruses isolated belonged to the 2.3.4.4b clade, which was the same clade circulating in Europe at that time [71]. The number of animals identified after the confirmation of the disease and the progression of the infection from the initially affected area to the entire farm suggested transmission amongst the minks. The source of infection is unknown; however, the epizootic situation of the region suggests that infected seabirds transmitted the virus, since there was an H5N1 wave in Galicia at that time and the minks were held in a partially open building [72]. The sequencing of the virus isolated from the mink samples showed several changes compared with the bird virus in PB2, PB1, PA, NA, NS2, M2, and PB1-F2. In particular, the mutation T271A in the PB2 gene was present in all the mink viruses sequenced. Whether this uncommon mutation arose de novo in the minks or was circulating in an unobserved avian virus is unknown. Indeed, from 2020 to 2022, this uncommon amino acid change (T271A) had been previously identified only in an H5N1 virus collected from an infected European polecat, in the Netherlands in March 2022 (GISAID EPI_ISL_13201074) [72]. The outbreak in minks is of particular concern; taking into account that minks have been shown to be permissive and susceptible to human and avian influenza, they could play a role similar to pigs in producing pandemic strains [73,74].

The H5N1 circulation in South America also featured mammalian infection. In January and February 2023, hundreds of sea lions were found dead or dying (634 animals) in Peru. The mass death of sea lions infected with H5N1 suggests that the virus may spread among mammals in the wild [75]. A report by the Agriculture Ministry of Peru on 4 March recorded at least 3487 South American sea lion deaths in the country that were associated with the HPAI A (H5N1) outbreak, representing about 3.3% of the total population in Peru [68,76]. All the Peruvian viruses belong to the 2.3.4.4b clade and do not contain the PB2 E627K or D701N mutations linked to mammalian host adaptation [77]. These three recent outbreaks in mammals as described above (USA, Spain, and Peru) could represent the first observations of mammal-to-mammal H5N1 transmission, which is another hallmark of the ongoing epizootic infection.

The risk of spread to humans increases with the occurrence of infections in domestic non-human mammals. This includes cats and dogs, which are pets that live in close contact with humans, frequently indoors. In April 2023, cases of HPAI H5N1 (2.3.4.4b) infections were reported in a domestic cat and five dogs living on a rural backyard poultry farm in Italy, where an HPAI H5N1 outbreak was detected [78]. In June 2023, the infection of 46 cats and a captive caracal was reported in Poland, 29 of which were positive for influenza H5N1 (clade 2.3.4.4b), 14 of which were euthanized, and a further 11 of which died. The sporadic infection of cats with H5N1 has been reported previously; however, this was the first report of a high number of cats infected with avian H5N1 over a wide geographical area within a country [79]. The more often the virus infects mammals, the more opportunity it has to evolve and adapt, increasing the chance of mammal-to-human as well as human-to-human transmission [80]. Fortunately, the circulating HPAI H5N1 viruses that affect birds and poultry, with spillover to mammals and humans, do not have the ability to efficiently bind to the receptors that predominate in the human upper respiratory tract [52].

A worrisome point is that the cases of humans infected by dairy cattle that were reported in the USA from March to May 2024 represent more likely evidence of mammal-to-human transmission [52]. The genome for the human isolate had three mutations: PB2 E627K, which is mentioned above; PB2 M631L; and PA K497R. The last two mutations were not observed in the cattle sequences and may be markers of predisposition to mammalian cell infection [81]. Up until June 2024, we observed not only bird-to-human and mammal-to-mammal transmission, but also mammal-to-human transmission. Human-to-human confirmed cases have not been reported yet; however, the new bird (poultry)-to-human and mammal (dairy cow)-to-human cases are of great concern [52].

### 3.4. Mutations of Concern

The following question has arisen: Would H5N1 Gs/GD-like variants evolve to infect mammals and humans due to mutations in the principal antigenic determinants?

Interestingly, the compiled information concerning natural H5N1 infections in mammals from 2020 through October 2023 shows several mutations in the HA [82]. Fortunately, the HA R226Q mutation—related to a binding enhancement to α2–6-linked sialic acids and a reduction in the pH threshold for membrane fusion [22]—was not detected [82]. In addition, other mutations, such as N158D/N224K/Q226L/T318I, were not identified either. These four mutations were enough to increase the respiratory droplet and air transmissibility of HPAI H5N1 A/Indonesia/5/2005 (clade 2.1.3.2) after a few passages in ferrets in gain-of-function experiments. Of note, this virus also included an additional amino acid change in the PB2 (T271A) protein, resulting in a virus with a high transmissibility [83].

Indeed, there is evidence of an association between the PB2 T271A mutation and the spillover of avian influenza viruses. Zhang and colleagues found that amino acid 271A of PB2 plays a key role in the virus’s acquisition of the mutation at position 226 of HA that confers human receptor recognition. The Q226R mutation of HA and the A271T mutation of PB2 together abolished the aerosol transmission of the virus A/Sichuan/1/2009 (H1N1) in ferrets [84]. Recently, the T271A mutation was detected in all the PB2 gene sequences of the H5N1 isolates collected from minks in the mass death event that occurred in Spain in 2022 [72] (see Section 3.3 above), with no mutations found in HA R226Q. The recombinant H5N1 virus derived from mink isolate A/mink/Spain/3691-8_22VIR10586-10/2022 (H5N1), clade 2.3.4.4b, which carried the adaptive mutation PB2 T271A, was assessed for its risk to humans. For this purpose, the potential of the virus to infect, cause disease, and transmit in ferrets was evaluated. Restori and colleagues showed that reversing the PB2 mutation (e.g., 271T) reduced the viral polymerase activity in mini-genome assays. Furthermore, introducing the PB2 A271T mutation reduced the mortality and resulted in a reduction in the number of respiratory contacts of ferrets that shed the virus in airborne transmission studies [85]. This was the first time that clade 2.3.4.4b exhibited direct-contact and airborne transmissibility in ferrets. The PB2 T271A mutation enhances the influenza A polymerase activity in mammalian host cells and mice, allowing for better virus replication [86]. This mutation is present in the avian-like PB2 gene of the H1N1pdm09 virus of swine origin; therefore, this amino acid change has potential public health implications [72]. Moreover, previous studies have shown that amino acid 271A of PB2 also plays a key role in the acquisition of this mutation by the virus at position 226 of HA that confers human receptor recognition [84].

Until now, the main mutations that contribute to mammalian infections were found in PB2 polymerase, E627K, D701N, and T271A. These mutations improve the ribonucleic acid polymerase activity and replication efficiency in mammalian cells, and they are considered to be early markers of mammalian adaptation [70], while the HA R226Q mutation has not been found [82]. The viral HA mediates the binding of the virus to cellular receptors and is the first virus protein that helps to define host range restrictions and susceptibility. Thus, HA-related changes in cellular receptor recognition – from α2-3 to α2-6 sialic acid receptor recognition – are known as potential features of avian viruses that have the ability to jump from birds to mammals. However, the evidence shows that spillover can also be caused by the mutation of genes other than HA, especially in PB2. This means that the change in the recognition of the host receptor by HA is an important, but not the sole, factor.

### 3.5. Gs/GD H5N1 Human Infections

Since the first human infection with avian influenza H5N1 documented in 1998, no proven human-to-human transmissions have been reported. In May 1997, a 3-year-old boy was found to be positive for the H5N1 virus; he subsequently died of respiratory failure on the 21st of May [87]. This was the first report of the H5N1 virus (A/Hong Kong/156/97, HK/97) infecting humans, related to the Gs/GD lineage. From 2003 to June 2024, 892 human cases of influenza A (H5N1) infections with 463 deaths were reported across 24 countries. The high lethality of H5N1 avian influenza (>50% in the last 20 years) associated with the severity of the disease makes it a great concern in the event that it becomes pandemic. Thirty new human infections were reported in the last three years (from July 2021 until June 2024), with most of them restricted to infections associated with contact with birds [52,53,88]. However, three human cases were reported in the USA following exposure to dairy cattle. Eight of the thirty total cases were fatal (Table 1): one in India, with a virus from H5 clade 2.3.2.1a [89]; one in China, with an H5 clade 2.3.4.4b virus [90]; and six related to clade 2.3.2.1c—one in Vietnam and five in Cambodia [52,91].

**Figure 1:**
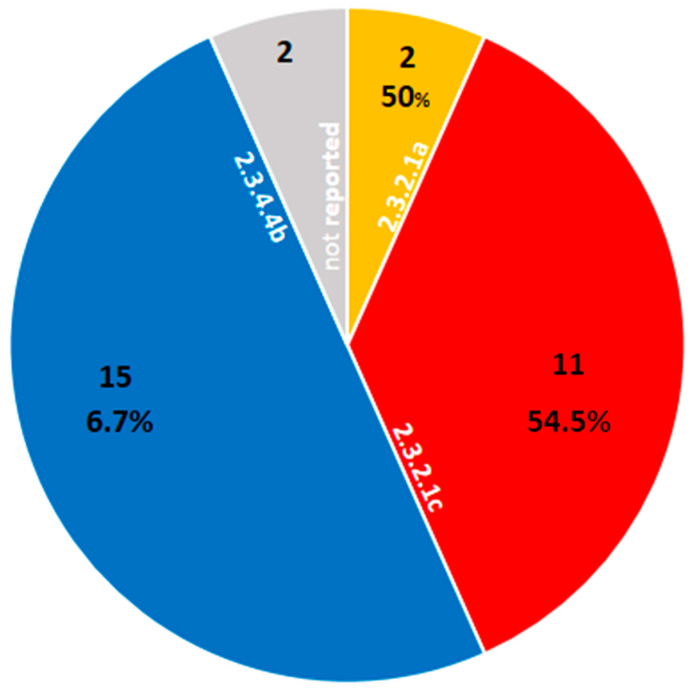
Influenza A (H5N1) lethality in humans by clade. Influenza A (H5N1) lethality in humans by clade (numbers in white). The colored sectors indicate the fraction of total cases (N = 30) in each clade: 2.3.2.1a (orange; N = 2), 2.3.2.1c (red; N = 11), 2.3.4.4b (blue; N = 15), and not reported (gray; N = 2). The percentages in each sector show the percent mortality relative to the number of cases in each clade. The period of analysis was from July 2021 to June 2024.

The lethality of H5N1 in humans from July 2021 up to June 2024 was 26.7% (eight deaths out of thirty cases). This value has been reduced almost by half compared to the HPAI H5N1 lethality since 2003—approximately 52%. This might indicate the circulation of a virus with a decreased virulence in humans. This observation makes sense, as clade 2.3.4.4b is less lethal (6.7%, with one death out of fifteen cases). The same situation was not observed for clades 2.3.2.1c, with a lethality of 54.5% (six deaths out of eleven cases), or for 2.3.2.1a (50%, with one death out of two cases) (Figure 1). Altogether, this indicates the following: (i) Clades 2.3.2.1a and 2.3.2.1c are highly lethal in humans, in contrast to 2.3.4.4b, which is more geographically dispersed and has the ability to infect different kinds of mammals and birds. (ii) The co-circulation of three different fatal H5N1 clades occurred in the same Asiatic region.

So far, in the ongoing epizootic, human H5N1 infections have been sporadic. No human-to-human transmissions have been observed yet, but the virus may acquire this feature in the near future [52]. Transmissibility among humans is a prerequisite for a novel influenza virus to become pandemic.

## 4. Choosing the Best CVVs for Vaccination against Circulating H5N1

To face a potential H5N1 pandemic, the World Health Organization (WHO) monitors the antigenic and genetic characteristics of zoonotic influenza A viruses and coordinates the development of CVVs by the WHO Collaborative Centers. CVVs encompassing clades 1 to 7 of different circulating antigenic prototypes have been developed by several laboratories. Some of these are ready to be produced and the reagents are available for vaccine potency tests [92,93]. The antigenic diversity among the clades complicates the control of H5N1 infections through vaccination, as different clades generally do not induce cross-neutralization [9].

A CVV against the wild type H5N1 virus should present the H5 HA antigen as the main target for the immune system, as the protein is responsible for influenza virus entry into the cell and it induces an immunodominant neutralizing antibody response [10]. The capacity to elicit these anti-H5 antibodies is tested through hemagglutination inhibition and microneutralization assays. Based on H5 HA, the selection of the best CVV for a pandemic situation passes through different levels of analysis: the CVV’s similarity to the circulating virus, the need to match different clades, the availability of the potency-testing reagents, the CVV’s similarity to pre-pandemic stockpiles and/or human licensed vaccines, and the existence of immune cross-reactions between the vaccines and the circulating clades are considered, among others. In this sense, an in silico analysis provides an opportunity to guide the selection of the CVVs, taking into account the variables mentioned above, and hence, it facilitates decision making regarding vaccine development for human use. This is especially important in a pandemic setting, when supplies may not be available and domestic manufacturing is crucial for active immunization. All these analyses are performed in order to reduce the chances of making an improper selection of an ill-suited CVV to fight against an ongoing epidemic as quickly as possible. The correct selection is further confirmed by clinical and field tests with the manufactured vaccine.

### 4.1. Selection of Circulating H5N1 Isolates for Analysis

The decision to develop a human vaccine is multifactorial; it takes into account the frequency of infection, the number of cases, the lethality, human infections, human deaths, mammal-to-human infections, and transmissions, among other factors. In order to prepare for pandemic influenza outbreaks, we focused on the clade circulating predominantly in the Americas and those viruses that have caused deaths in humans. Our analyses included 70 amino acid sequences from isolates circulating in North America from January to December 2022—sixty-nine sequences collected in the USA and one in Mexico, with one human case collected in the USA on April 20th [59]. The sequences were aligned and the consensus sequence was identical to the GenBank UWI70278.1 H5, which was part of the collection [60]. Upon selecting the best CVV, it is important to consider not only its similarity to the most frequent circulating variant or clade, but also its antigenic relationship with variants of clinical relevance. Thus, from the 12 new H5N1 human cases reported over two years (July 2021 to May 2023) (Table 1), we selected one sequence from each clade that caused deaths or critical illnesses from different regions of the world. They were the GenBank UDV79400 (A/India/SARI-4571/2021, collected from an 11-year-old boy who died in India on 12 July 2021 [clade 2.3.2.1a]), GISAID EPI2419700 (A/Cambodia/NPH230032/2023, collected from an 11-year-old girl who died on February 23 2023 in southern Cambodia [clade 2.3.2.1c]), and GISAID EPI2510183 (A/Chile/25945/2023, collected from a 53-year-old man with a critical illness in Chile on March 24, 2023 [clade 2.3.4.4b]) H5 sequences [90]. The last sequence was a representative of the South American sequences and was identical to H5 GenBank WDE94949.1 (A/Pelican/CHL/227087-1/2022), a representative sequence from the analysis of the H5 sequences of 12 isolates collected from wild birds in Chile in December 2022. The Chilean bird sequence shared a >99% identity with the H5 sequences of the isolates that circulated in the Americas from January to December 2022 [60].

Thus, our analysis tagged the following: (1) two bird sequences, GenBank WAH70677.1 (A/Pelecanus occidentalis/Venezuela/Pel4S4/2022) from a Venezuelan isolate and GenBank UWI70278.1 (A/common eiders/Maine/W22-481A/2022), a representative of the 70 North American sequences, and (2) the three aforementioned human sequences.

### 4.2. Relationship between Isolates and CVVs

The analysis (using phylogenetic and BLAST tools) of the H5 amino acid sequences allowed for an understanding of the relationship among H5N1 isolates and the antigenic prototype of CVVs from different clades (see Table 2). By comparing the H5 amino acid sequences of the H5N1 viral isolates mentioned above (UWI70278 [2.3.4.4b], UDV79400 [2.3.2.1a], EPI2419700 [2.3.2.1c], and EPI2510183 [2.3.4.4b]) to the H5 HA of the 36 H5Nx CVV antigenic prototypes, it was observed that IDCDC-RG71A (A/Astrakhan/3212/2020 (H5N8)) would be the best choice for a monovalent vaccine for clade 2.3.4.4b isolates, which are distinct from the Indian (2.3.2.1a) and Cambodian (2.3.2.1c) isolates. Indeed, the best CVV antigenic prototype for 2.3.2.1a would be SJ001 (A/duck/Bangladesh/19097/2013 (H5N1)), while for 2.3.2.1c, it would be NIBRG-301 (A/duck/Vietnam/NCVD-1584/2012 (H5N1)) [60]. Moreover, considering the current licensed H5N1 vaccines approved for pandemic use [94,95], it is also clear that they are not the best choice in the case of outbreaks related to H5N1 clades 2.3.2.1a, 2.3.2.1c, or 2.3.4.4b. They were developed to immunize against the H5N1 virus related to clades 1 (A/Vietnam/1194/2004 (H5N1) [NIBRG-14]), 2.1.3.2 (A/Indonesia/5/2005 (H5N1) [IDCDC-RG2]), and 2.2.1 (A/turkey/Turkey/1/2005 (H5N1) [NIBRG-23]), which are different from the clades of the current epizootic situation. By analyzing the globular domain sequence of H5 HA, a higher level of identity was observed for the IDCDC-RG71A CVV to H5N1 isolates from the USA and Chile (amino acid sequence identity > 98%), for the NIBRG-301 CVV to the Cambodian isolate (95–96% of amino acid sequence identity), and for the SJ001 CVV to the Indian isolate (95–96% of amino acid sequence identity). This contrasts with the relatively lower percentage of amino acid sequence identity when comparing the H5 HA of licensed vaccines (84–91% identity) [60]. The same conclusion can be drawn from the phylogenetic analysis: licensed H5N1 vaccines belong to a different group than the CVVs IDCDC-RG71A, SJ001, and NIBRG-301, showing that these new CVVs are more suitable for vaccine development against the current wave of H5N1 [60].

In addition, potency-testing reagents were prepared against CVVs, allowing for the immediate production and dosing of these new H5Nx vaccine prototypes for pre-clinical and clinical studies. Table 2 displays a list of the H5Nx CVVs and the potency-testing reagents for clades 2.3.2.1a, 2.3.2.1 c, and 2.3.4.4b [92,93,96]. Thus, H5Nx strains bearing H5 amino acid sequences related to circulating H5 clade 2.3.4.4b in the Americas constitute antigenic prototypes that can be used as CVVs for vaccine development against H5N1 as well as H5N6 [44], H5N8 [49], or any other H5Nx of the same clade. In this case, IDCDC-RG71A and CBER-RG8A CVVs were developed, both of which were derived from A/Astrakhan/3212/2020 (H5N8). Reference antigens available for these CVVs comprise IDCDC-RG71A, SeqHSsYs22_09.5a, and CBER-RG8A from CBER/FDA (USA), TGA (Australia), and MHRA (UK). Other CVVs for H5 clade 2.3.4.4b are also available (Table 2), including IDCDC-RG78A (A/American wigeon/South Carolina/22- 000345-001/2021-like (H5N1)) and NIID-002 (A/Ezo red fox/Hokkaido/1/2022-like (H5N1)), but neither reference antigens nor specific antisera have been produced. Therefore, the sheep antisera produced against H5N8 A/Astrakhan/3212/2020 and reference antigens that belong to the same clade (2.3.4.4b) should be used for vaccine calibrations. For the other H5N1 viruses circulating in Asia that are associated with human deaths, CVVs for the H5 clades 2.3.2.1a (SJ001 (A/duck/Bangladesh/19097/2013 (H5N1)) and 2.3.2.1c (NIBRG-301 (A/duck/Vietnam/NCVD-1584/2012 (H5N1)) are indicated, since the reference antigens and antisera are available (Table 2).

The selection or identification of the best-matched CVV was carried out during the ongoing H5N1 epizootic. The spread of the virus in a large mass of animals (birds and non-human mammals) might give rise to new strains with divergent H5 sequences. In this case, monitoring the similarity of the H5 sequences between circulating isolates and available and stockpiled CVVs, as well as seasonal or human-licensed vaccines, is crucial for preliminarily assessing the antigenic relatedness. According to the influenza risk assessment tool, this is one of the ten issues that are considered when assessing the risk of a non-circulating virus in humans becoming pandemic [97]. Considering clade 2.3.4.4b’s global distribution, its broad host range, and the detection of some mutations associated with mammalian adaptation, clade 2.3.4.4b might be considered as the most likely emergent pandemic virus. Nevertheless, it is not possible to predict for sure if a pandemic will occur, and if so, which virus will be responsible. Indeed, we know which strains and clades are circulating, and based on risk analyses, actions can be planned. Preparedness against potential pandemic H5N1 or H5Nx circulating strains for clades 2.3.2.1a, 2.3.2.1c, and 2.3.4.4b can be achieved through the production of active substances to formulate vaccines for use in case of a pandemic associated with these clades, due to the availability of related CVVs, reference antigens, and antisera reagents (Table 2). In the case of H5 clade 2.3.4.4b, the possible CVVs comprise the antigenic prototypes A/Astrakhan/3212/2020 (H5N8), A/American wigeon/South Carolina/22- 000345-001/2021-like (H5N1), and A/Ezo red fox/Hokkaido/1/2022-like (H5N1). The CVV for A/American wigeon/South Carolina/22- 000345-001/2021-like antigenic prototype is available through the CDC (USA) and A/Ezo red fox/Hokkaido/1/2022-like (H5N1) is available from NIID (Japan), although the reference reagents are not. In these cases, the reference reagents and antisera from antigen prototype A/Astrakhan/3212/2020 (H5N8) CVVs might be used to calibrate the vaccine potency. For clade 2.3.2.1a, the indicated CVV is related to antigen prototype A/duck/Bangladesh/19097/2013, and for clade 2.3.1.1c, it is related to A/duck/Vietnam/NCVD-1584/2012.

## 5. Concluding Remarks

The mass death events in mammals reinforce the need to monitor mutations emerging in the principal antigenic determinants (hemagglutinin, polymerase, and neuraminidase) and assess the mammalian host adaptations. H5N1 viruses related to clades 2.3.4.4b, 2.3.2.1a, and 2.3.2.1c were responsible for the recent human fatal cases, with clade 2.3.4.4b predominantly infecting and causing death in birds and mammals in the ongoing epidemic. Although it is recognized that the H5N1, H5N2, H5N3, H5N4, and H5N5 variants (bearing the 2.3.4.4b clade) have the same origin, with H5N8 reassorted to the avian influenza virus, H5N1 is now the most common strain responsible for global avian influenza outbreaks and distribution [53]. H5N1 has been reported in a wide range of host species, including wild birds, domestic poultry, wild mammals [51], dogs [78], cats [79], and cows [52], indicating an increased risk for virus incursion and adaptation into new hosts. Even though other clade 2.3.4.4b-related CVVs are available, the IDCDC-RG71A and CBER-RG8A (A/Astrakhan/3212/2020 (H5N8)) CVVs are currently the most suitable to be used for vaccine development against this clade. These are CVVs whose antigenic prototype fits for the H5 2.3.4.4b circulating clade, and reference antigens and antisera have been produced against the same CVVs. Therefore, these would be the most adequate monovalent vaccines to be launched in the case of a pandemic caused by the current HPAI H5N1 clade 2.3.4.4b virus, which is predominantly circulating. Interestingly, the antigenic prototype could also be a suitable CVV against circulating H5Nx of the same clade (2.3.4.4b). The entry of H5N1 clade 2.3.4.4b into the Americas and, especially, in South America, makes the H5N1 pandemic risk more eminent. On the other hand, in spite of the 2.3.4.4b clade’s global predominance, other H5 clades are also circulating in the same Asiatic region and causing human deaths. Upon the selection of the best CVV, another aspect to consider is its antigenic relationship with clinical variants of H5N1 viruses. Our analyses targeted those clades that have been reported to cause deaths in humans over the last three years, although with a more restricted distribution. Therefore, due to recent fatal cases in the Asiatic region, other CVVs are also of interest: i.e., SJ001 (A/duck/Bangladesh/19097/2013 (H5N1)) and NIBRG-301 (A/duck/Vietnam/NCVD-1584/2012 (H5N1)) which are related to the 2.3.2.1a and 2.3.2.1c clades, respectively. Fortunately, we have available CVVs and reference reagents (antigens and antisera) for vaccine calibrations against these clades (2.3.4.4b, 2.3.2.1a, and 2.3.2.1c).

New technologies might lessen the vaccine production time, even to 100 days, contributing to the achievement of the “100 Days Mission” goal, where governments, academia, industries, philanthropies, and civil societies gather to make safe effective vaccines, therapeutics, and diagnostics within 100 days of pandemic pathogen identification [98]. Previous pandemics and epidemics (influenza, Zika, Ebola, monkeypox, and Marburg) and, certainly, the recent devastating COVID-19 pandemic have prompted this initiative to mitigate and stop future pandemics in a shorter time [99]. Considering the lessons learned from past and recent pandemics, alerts are in the air, and we must be prepared for an H5N1 pandemic.

## Figures and Tables

**Table 1:** Global reported A (H5N1) human cases, July 2021 through 4 June 2024.

Virus Clade by Sequencing or Associated Poultry Outbreaks	Month of Illness Onset or Case Detection	Death	Country of Case
Clade 2.3.2.1a	July 2021	yes	India
	March 2024	no	Australia (after travel to India)
Clade 2.3.2.1c	February 2023	yes	Cambodia
	February 2023	no	
	October 2023	yes	
	October 2023	yes	
	November 2023	yes	
	November 2023	no	
	January 2024	no	
	January 2024	no	
	January 2024	yes	
	February 2024	no	
	March 2024	yes	Vietnam
Clade 2.3.4.4b	March 2023	no	Chile
	September 2022	yes	China
	January 2023	no	
	December 2022	no	Ecuador
	September 2022	no	Spain
	October 2022	no	
	January 2022	no	United Kingdom
	May 2023	no	
	May 2023	no	
	July 2023	no	
	July 2023	no	
	April 2022	no	United States
	March 2024	no	
	May 2024	no	
	May 2024	no	
Not reported	October 2022	no	Vietnam
	February 2024	no	Cambodia

Adapted from https://www.cdc.gov/bird-flu/php/technical-report/h5n1-06052024.html (accessed on 26 June 2024) [52].

**Table 2:** Status of development and availability of H5Nx CVVs and potency testing reagents from clades 2.3.2.1a, 2.3.2.1c, and 2.3.4.4.b.

		Candidate Vaccine Virus (CVV)				Reference Antigen (Freeze Dried)					Sheep Antisera	
Clade	Subtype	Antigenic Prototype	Candidate Vaccine Virus	Developing Institute	Available From	CVV Starting Material		Ref Ag Lot Number	Unitage (µgHA/mL)	Available From	Order Lot Number	Available From
2.3.2.1a	H5N1	A/Hubei/1/2010	Wild type virus		WHO CCs							
			IDCDC-RG30 *	CCDC, China CDC, USA	CCDC, China CDC, USA	N/A		N/A	N/A	N/A	N/A	N/A
		A/duck/Bangladesh/19097/2013	Wild type virus		WHO CCs							
			SJ001 *	SJCRH, USA	SJCRH, USA	N/A		H5-Ag1409 (cell)	88	CBER/FDA, USA	H5-Ab-1513	CBER/FDA, USA
		A/duck/Bangladesh/17D1012/2018	IDCDC-RG63A *	CDC, USA	CDC, USA	N/A		N/A	N/A	N/A	N/A	N/A
2.3.2.1c	H5N1	A/duck/Vietnam/NCVD-1584/2012	Wild type virus		WHO CCs							
			NIBRG-301 *	MHRA, UK	MHRA, UK	NIBRG-301		17/142	47	MHRA, UK	14/318	MHRA, UK
	H5N1	A/American wigeon/South Carolina/22- 000345-001/2021-like		Wild type virus								
				IDCDC-RG78A *	CDC, USA	CDC, USA	N/A	N/A	N/A	N/A	N/A	N/A
		A/American wigeon/South Carolina/AH0195145/2021		SeqHSSVR02	CBER/FDA, USA	NA	SeqHSSVR02	H5-Ag2311 (cell) **	96	CBER/FDA, USA	N/A	N/A
		A/Ezo red fox/Hokkaido/1/2022-like		NIID-002 *	NIID, Japan	NIID, Japan	N/A	N/A	N/A	N/A	N/A	N/A
		A/chicken/Ghana/AVL-76321VIR7050- 39/2021-like		N/A	CDC, USA	Pending	N/A	N/A	N/A	N/A	N/A	N/A
2.3.4.4b	H5N6	A/Fujian-Sanyuan/21099/2017-like		N/A	CCDC, China	Pending	N/A	N/A	N/A	N/A	N/A	N/A
	H5N8	A/Astrakhan/3212/2020		IDCDC-RG71A *	CDC, USA	CDC, USA	IDCDC-RG71A	H5-Ag2301 (egg)	62	CBER/FDA, USA	H5-Ab-2313	CBER/FDA, USA
				CBER-RG8A *	CBER/FDA, USA	CBER/FDA, USA	SeqHSsYs22_09.5a	H5-Ag2213 (cell)	98			
							CBER-RG8A	2023/144B (egg)	72	TGA, Australia		
								23/178	Lyo ***	MHRA, UK	23/224	MHRA, UK

* CVV that can be handled under BSL-2 enhanced containment; ** calibrated using anti-A/Astrakhan/3212/2020 HA antisera, CBER Lot #H5-Ab-2313; *** net weight: 1g; and N/A—not available. Adapted from https://cdn.who.int/media/docs/default-source/influenza/cvvs/cvv-zoonotic-northern-hemisphere-2024-2025/h5n1_summary_a_h5n1_cvv_20240223.pdf?sfvrsn=2d559bc4_5&download=true (accessed on 26 June 2024) [92] and https://cdn.who.int/media/docs/default-source/influenza/cvvs/cvv-zoonotic---southern-hemisphere-2024/summary_a_h5_cvv_sh24.pdf?sfvrsn=a3dc6046_4&download=true (accessed on 26 June 2024) [93].

## Data Availability

The data that support the findings of this study are openly available in the repository “Selecting the best candidate vaccine virus for circulating H5N1” at https://doi.org/10.6084/m9.figshare.24324523 [60].

## References

[1] Past Pandemics. https://www.cdc.gov/flu/pandemic-resources/basics/past-pandemics.html.

[2] 1918 Pandemic (H1N1 Virus). https://www.cdc.gov/flu/pandemic-resources/1918-pandemic-h1n1.html.

[3] Jester B.J., Uyeki T.M., Patel A., Koonin L., Jernigan D.B. (2018). 100 Years of Medical Countermeasures and Pandemic Influenza Preparedness. Am. J. Public Heal..

[4] 2009 H1N1 Pandemic (H1N1pdm09 Virus). https://www.cdc.gov/flu/pandemic-resources/2009-h1n1-pandemic.html.

[5] 1957–1958 Pandemic (H2N2 Virus). https://www.cdc.gov/flu/pandemic-resources/1957-1958-pandemic.html.

[6] 1968 Pandemic (H3N2 Virus). https://www.cdc.gov/flu/pandemic-resources/1968-pandemic.html.

[7] Wille M., Barr I.G. (2022). Resurgence of avian influenza virus. Science.

[8] Fouchier R.A.M., Munster V., Wallensten A., Bestebroer T.M., Herfst S., Smith D., Rimmelzwaan G.F., Olsen B., Osterhaus A.D.M.E. (2005). Characterization of a Novel Influenza A Virus Hemagglutinin Subtype (H16) Obtained from Black-Headed Gulls. J. Virol..

[9] Tosh C. (2014). Evolution and Spread of Avian Influenza H5N1 Viruses. Adv. Anim. Veter. Sci..

[10] Petrova V.N., Russell C.A. (2017). Erratum: The evolution of seasonal influenza viruses. Nat. Rev. Microbiol..

[11] Alexander D.J. (2007). An overview of the epidemiology of avian influenza. Vaccine.

[12] Gischke M., Bagato O., Breithaupt A., Scheibner D., Blaurock C., Vallbracht M., Karger A., Crossley B., Veits J., Böttcher-Friebertshäuser E. (2021). The role of glycosylation in the N-terminus of the hemagglutinin of a unique H4N2 with a natural polybasic cleavage site in virus fitness In Vitro and In Vivo. Virulence.

[13] Vey M., Schäfer W., Reis B., Ohuchi R., Britt W., Garten W., Klenk H.-D., Radsak K. (1995). Proteolytic processing of human cytomegalovirus glycoprotein B (gpUL55) is mediatedby the human endoprotease furin. Virology.

[14] Bertram S., Glowacka I., Steffen I., Kühl A., Pöhlmann S. (2010). Novel insights into proteolytic cleavage of influenza virus hemagglutinin. Rev. Med. Virol..

[15] Böttcher-Friebertshäuser E., Klenk H.-D., Garten W. (2013). Activation of influenza viruses by proteases from host cells and bacteria in the human airway epithelium. Pathog. Dis..

[16] Izaguirre G. (2019). The Proteolytic Regulation of Virus Cell Entry by Furin and Other Proprotein Convertases. Viruses.

[17] Banks J., Speidel E.S., Moore E., Plowright L., Piccirillo A., Capua I., Cordioli P., Fioretti A., Alexander D.J. (2001). Changes in the haemagglutinin and the neuraminidase genes prior to the emergence of highly pathogenic H7N1 avian influenza viruses in Italy. Arch. Virol..

[18] Suarez D.L., Senne D.A., Banks J., Brown I.H., Essen S.C., Lee C.-W., Manvell R.J., Mathieu-Benson C., Moreno V., Pedersen J.C. (2004). Recombination Resulting in Virulence Shift in Avian Influenza Outbreak, Chile. Emerg. Infect. Dis..

[19] Khatchikian D., Orlich M., Rott R. (1989). Increased viral pathogenicity after insertion of a 28S ribosomal RNA sequence into the haemagglutinin gene of an influenza virus. Nature.

[20] Orlich M., Gottwald H., Rott R. (1994). Nonhomologous Recombination between the Hemagglutinin Gene and the Nucleoprotein Gene of an Influenza Virus. Virology.

[21] Laleye A.T., Abolnik C. (2020). Emergence of highly pathogenic H5N2 and H7N1 influenza A viruses from low pathogenic precursors by serial passage in ovo. PLoS ONE.

[22] Skehel J.J., Wiley D.C. (2000). Receptor Binding and Membrane Fusion in Virus Entry: The Influenza Hemagglutinin. Annu. Rev. Biochem..

[23] FAO H5N1 HPAI Global Overview: January–March 2012. https://bit.ly/3L2TGmd.

[24] https://www.who.int/publications/m/item/influenza-a(h5n1)-highly-pathogenic-avian-influenza-timeline-of-major-events.

[25] Xu X., Subbarao K., Cox N.J., Guo Y. (1999). Genetic Characterization of the Pathogenic Influenza A/Goose/Guangdong/1/96 (H5N1) Virus: Similarity of Its Hemagglutinin Gene to Those of H5N1 Viruses from the 1997 Outbreaks in Hong Kong. Virology.

[26] Data, Disease and Diplomacy: GISAID’s Innovative Contribution to Global Health. https://gisaid.org/.

[27] Guan Y., Peiris J.S.M., Lipatov A.S., Ellis T.M., Dyrting K.C., Krauss S., Zhang L.J., Webster R.G., Shortridge K.F. (2002). Emergence of multiple genotypes of H5N1 avian influenza viruses in Hong Kong SAR. Proc. Natl. Acad. Sci. USA.

[28] Li K.S., Guan Y., Wang J., Smith G.J.D., Xu K.M., Duan L., Rahardjo A.P., Puthavathana P., Buranathai C., Nguyen T.D. (2004). Genesis of a highly pathogenic and potentially pandemic H5N1 influenza virus in eastern Asia. Nature.

[29] Guan Y., Poon L.L.M., Ellis T.M., Lim W., Lipatov A.S., Chan K.H., Sturm-Ramirez K.M., Cheung C.L., Leung Y.H.C., Yuen K.Y. (2004). H5N1 influenza: A protean pandemic threat. Proc. Natl. Acad. Sci. USA.

[30] WHO/OIE/FAO/H5N1 Evolution Working Group (2008). Toward a Unified Nomenclature System for Highly Pathogenic Avian Influenza Virus (H5N1). Emerg. Infect. Dis..

[31] (2011). WHO/OIE/FAO H5N1 Evolution Working Group Continued evolution of highly pathogenic avian influenza A (H5N1): Updated nomenclature. Influenza Other Respir. Viruses.

[32] Smith G.J.D., Donis R.O., World Health Organization/World Organisation for Animal Health/Food and Agriculture Organization (WHO/OIE/FAO) H5 Evolution Working Group (2015). Nomenclature updates resulting from the evolution of avian influenza A (H5) virus clades 2.1.3.2a, 2.2.1, and 2.3.4 during 2013–2014. Influenza Other Respir. Viruses.

[33] Chen H., Smith G.J.D., Zhang S.Y., Qin K., Wang J., Li K.S., Webster R.G., Peiris J.S.M., Guan Y. (2005). H5N1 virus outbreak in migratory waterfowl. Nature.

[34] Liu J., Xiao H., Lei F., Zhu Q., Qin K., Zhang X.-L., Zhao D., Wang G., Feng Y., Ma J. (2005). Highly Pathogenic H5N1 Influenza Virus Infection in Migratory Birds. Science.

[35] Couacy-Hymann E., Kouakou V.A., Aplogan G.L., Awoume F., Kouakou C.K., Kakpo L., Sharp B.R., McClenaghan L., McKenzie P., Webster R.G. (2012). Surveillance for Influenza Viruses in Poultry and Swine, West Africa, 2006–2008. Emerg. Infect. Dis..

[36] Harfoot R., Webby R.J. (2017). H5 influenza, a global update. J. Microbiol..

[37] Reid S.M., Shell W.M., Barboi G., Onita I., Turcitu M., Cioranu R., Marinova-Petkova A., Goujgoulova G., Webby R.J., Webster R.G. (2010). First Reported Incursion of Highly Pathogenic Notifiable Avian Influenza A H5N1 Viruses from Clade 2.3.2 into European Poultry. Transbound. Emerg. Dis..

[38] Evolution of the Influenza A(H5) Haemagglutinin: WHO/OIE/FAO H5 Working Group Reports a New Clade Designated 2.3.4.4. https://www.who.int/publications/m/item/evolution-of-the-influenza-a(h5)-haemagglutinin-who-oie-fao-h5-working-group-reports-a-new-clade-designated-2.3.4.4.

[39] Ip H.S., Torchetti M.K., Crespo R., Kohrs P., DeBruyn P., Mansfield K.G., Baszler T., Badcoe L., Bodenstein B., Shearn-Bochsler V. (2015). Novel Eurasian Highly Pathogenic Avian Influenza A H5 Viruses in Wild Birds, Washington, USA, 2014. Emerg. Infect. Dis..

[40] Pasick J., Berhane Y., Joseph T., Bowes V., Hisanaga T., Handel K., Alexandersen S. (2015). Reassortant Highly Pathogenic Influenza A H5N2 Virus Containing Gene Segments Related to Eurasian H5N8 in British Columbia, Canada, 2014. Sci. Rep..

[41] Gu M., Zhao G., Zhao K., Zhong L., Huang J., Wan H., Wang X., Liu W., Liu H., Peng D. (2013). Novel Variants of Clade 2.3.4 Highly Pathogenic Avian Influenza A(H5N1) Viruses, China. Emerg. Infect. Dis..

[42] Sobolev I., Sharshov K., Dubovitskiy N., Kurskaya O., Alekseev A., Leonov S., Yushkov Y., Irza V., Komissarov A., Fadeev A. (2021). Highly Pathogenic Avian Influenza A(H5N8) Virus Clade 2.3.4.4b, Western Siberia, Russia, 2020. Emerg. Infect. Dis..

[43] Kupferschmidt K. (2023). Bird flu spread between mink is a ‘warning bell’. Science.

[44] Lewis N.S., Banyard A.C., Whittard E., Karibayev T., Al Kafagi T., Chvala I., Byrne A., (Akberovna) S.M., King J., Harder T. (2021). Emergence and spread of novel H5N8, H5N5 and H5N1 clade 2.3.4.4 highly pathogenic avian influenza in 2020. Emerg. Microbes Infect..

[45] El-Shesheny R., Kandeil A., Mostafa A., Ali M.A., Webby R.J. (2020). H5 Influenza Viruses in Egypt. Cold Spring Harb. Perspect. Med..

[46] Baek Y.-G., Lee Y.-N., Lee D.-H., Shin J.-I., Lee J.-H., Chung D.H., Lee E.-K., Heo G.-B., Sagong M., Kye S.-J. (2021). Multiple Reassortants of H5N8 Clade 2.3.4.4b Highly Pathogenic Avian Influenza Viruses Detected in South Korea during the Winter of 2020–2021. Viruses.

[47] Disease Outbreak News. Human Infection with Avian Influenza A (H5N8)—Russian Federation. https://www.who.int/emergencies/disease-outbreak-news/item/2021-DON313.

[48] King J., Harder T., Globig A., Stacker L., Günther A., Grund C., Beer M., Pohlmann A. (2022). Highly pathogenic avian influenza virus incursions of subtype H5N8, H5N5, H5N1, H5N4, and H5N3 in Germany during 2020-21. Virus Evol..

[49] Gu W., Shi J., Cui P., Yan C., Zhang Y., Wang C., Zhang Y., Xing X., Zeng X., Liu L. (2022). Novel H5N6 reassortants bearing the clade 2.3.4.4b HA gene of H5N8 virus have been detected in poultry and caused multiple human infections in China. Emerg. Microbes Infect..

[50] Bevins S.N., Shriner S.A., Cumbee J.C., Dilione K.E., Douglass K.E., Ellis J.W., Killian M.L., Torchetti M.K., Lenoch J.B. (2022). Intercontinental Movement of Highly Pathogenic Avian Influenza A(H5N1) Clade 2.3.4.4 Virus to the United States, 2021. Emerg. Infect. Dis..

[51] Adlhoch C., Fusaro A., Gonzales J.L., Kuiken T., Marangon S., Niqueux E., Staubach C., Terregino C., Aznar I., European Food Safety Authority, European Centre for Disease Prevention and Control, European Union Reference Laboratory for Avian Influenza (2022). Avian influenza overview December 2021–March 2022. EFSA J..

[52] Technical Report: June 2024 Highly Pathogenic Avian Influenza A(H5N1) Viruses. https://www.cdc.gov/bird-flu/php/technical-report/h5n1-06052024.html?CDC_AAref_Val=https://www.cdc.gov/flu/avianflu/spotlights/2023-2024/h5n1-technical-report-06052024.htm.

[53] Fusaro A., Gonzales J.L., Kuiken T., Mirinavičiūtė G., Niqueux E., Ståhl K., Staubach C., EFSA (European Food Safety Authority), ECDC (European Centre for Disease Prevention and Control), EURL (European Union Reference Laboratory for Avian Influenza) (2024). Scientific report: Avian influenza overview December 2023–March 2024. EFSA J..

[54] High Pathogenicity Avian Influenza (HPAI)-Situation Report. https://www.woah.org/app/uploads/2023/03/hpai-situation-report-20230311.pdf.

[55] High Pathogenicity Avian Influenza (HPAI)-Situation Report 34. https://www.woah.org/en/document/high-pathogenicity-avian-influenza-hpai-situation-report-34.

[56] Xu W., Berhane Y., Dubé C., Liang B., Pasick J., VanDomselaar G., Alexandersen S. (2016). Epidemiological and Evolutionary Inference of the Transmission Network of the 2014 Highly Pathogenic Avian Influenza H5N2 Outbreak in British Columbia, Canada. Sci. Rep..

[57] Caliendo V., Lewis N.S., Pohlmann A., Baillie S.R., Banyard A.C., Beer M., Brown I.H., Fouchier R.A.M., Hansen R.D.E., Lameris T.K. (2022). Transatlantic spread of highly pathogenic avian influenza H5N1 by wild birds from Europe to North America in 2021. Sci. Rep..

[58] The Influenza Virus Resource at the National Center for Biotechnology Information. https://www.ncbi.nlm.nih.gov/genomes/FLU/Database/nph-select.cgi?go=database.

[59] Epidemiological Alert: Outbreak of avian Influenza caused by influenza A (H5N1) in the Region of the Americas. https://www.paho.org/en/documents/epidemiological-alert-outbreaks-avian-influenza-caused-influenza-ah5n1-region-americas.

[60] Medina Y., Akamatsu M., Oliveira R. (2023). Selecting the best candidate vaccine virus for potential pandemic H5N1 currently in circulation. figshare. Dataset.

[61] Avian Influenza Virus Type A (H5N1) in U.S. Dairy Cattle. https://www.avma.org/resources-tools/animal-health-and-welfare/animal-health/avian-influenza/avian-influenza-virus-type-h5n1-us-dairy-cattle.

[62] NOTA TÉCNICA No 11/2023/DSA/SDA/MAPA. https://www.agricultura.rs.gov.br/upload/arquivos/202305/16091258-h5n1-brasil-port-eng-esp.pdf.

[63] Hurtado R., de Azevedo-Júnior S.M., Vanstreels R.E.T., Fabrizio T., Walker D., Rodrigues R.C., Seixas M.M.M., de Araújo J., Thomazelli L.M., Ometto T.L. (2016). Surveillance of Avian Influenza Virus in Aquatic Birds on the Brazilian Amazon Coast. Ecohealth.

[64] Imai M., Kawaoka Y. (2012). The role of receptor binding specificity in interspecies transmission of influenza viruses. Curr. Opin. Virol..

[65] Kuchipudi S.V., Nelli R.K., Gontu A., Satyakumar R., Nair M.S., Subbiah M. (2021). Sialic Acid Receptors: The Key to Solving the Enigma of Zoonotic Virus Spillover. Viruses.

[66] Ito T., Couceiro J.N.S.S., Kelm S., Baum L.G., Krauss S., Castrucci M.R., Donatelli I., Kida H., Paulson J.C., Webster R.G. (1998). Molecular Basis for the Generation in Pigs of Influenza A Viruses with Pandemic Potential. J. Virol..

[67] Hervé S., Schmitz A., Briand F.-X., Gorin S., Quéguiner S., Niqueux E., Paboeuf F., Scoizec A., Le Bouquin-Leneveu S., Eterradossi N. (2021). Serological Evidence of Backyard Pig Exposure to Highly Pathogenic Avian Influenza H5N8 Virus during 2016–2017 Epizootic in France. Pathogens.

[68] Adlhoch C., Fusaro A., Gonzales J.L., Kuiken T., Marangon S., Mirinaviciute G., Niqueux E., Stahl K., EFSA, ECDC, EURL (European Food Safety Authority, European Centre for Disease Prevention and Control, European Ref-erence Laboratory) (2023). Scientific report: Avian influenza overview December 2022–March 2023. EFSA J..

[69] Puryear W., Sawatzki K., Hill N., Foss A., Stone J.J., Doughty L., Walk D., Gilbert K., Murray M., Cox E. (2023). Highly Pathogenic Avian Influenza A(H5N1) Virus Outbreak in New England Seals, United States. Emerg. Infect. Dis..

[70] Gao Y., Zhang Y., Shinya K., Deng G., Jiang Y., Li Z., Guan Y., Tian G., Li Y., Shi J. (2009). Identification of Amino Acids in HA and PB2 Critical for the Transmission of H5N1 Avian Influenza Viruses in a Mammalian Host. PLoS Pathog..

[71] Adlhoch C., Fusaro A., Gonzales J.L., Kuiken T., Marangon S., Niqueux E., Staubach C., EFSA (European Food Safety Authority), ECDC (European Centre for Disease Prevention and Control), EURL (European Reference Laboratory for Avian Influenza) (2023). Scientific report: Avian influenza overview September–December 2022. EFSA J..

[72] Agüero M., Monne I., Sánchez A., Zecchin B., Fusaro A., Ruano M.J., Arrojo M.d.V., Fernández-Antonio R., Souto A.M., Tordable P. (2023). Highly pathogenic avian influenza A(H5N1) virus infection in farmed minks, Spain, October 2022. Eurosurveillance.

[73] Crisci E., Mussá T., Fraile L., Montoya M. (2013). Review: Influenza virus in pigs. Mol. Immunol..

[74] Sun H., Li F., Liu Q., Du J., Liu L., Sun H., Li C., Liu J., Zhang X., Yang J. (2021). Mink is a highly susceptible host species to circulating human and avian influenza viruses. Emerg. Microbes Infect..

[75] Mass Death of Sea Lions from Bird Flu Suggests Virus may be Spreading between Mammals in the Wild. https://english.elpais.com/science-tech/2023-02-15/mass-death-of-sea-lions-from-bird-flu-suggests-virus-may-be-spreading-between-mammals-in-the-wild.html.

[76] First Birds, Now Mammals: How H5N1 is Killing Thousands of Sea Lions in Peru. https://www.theguardian.com/environment/2023/mar/21/bird-flu-peru-sea-lions-suffer-death-beach-aoe-h5n1.

[77] Leguia M., Garcia-Glaessner A., Muñoz-Saavedra B., Juarez D., Barrera P., Calvo-Mac C., Jara J., Silva W., Ploog K., Amaro L. (2023). Highly pathogenic avian influenza A (H5N1) in marine mammals and seabirds in Peru. Nat. Commun..

[78] Moreno A., Bonfante F., Bortolami A., Cassaniti I., Caruana A., Cottini V., Cereda D., Farioli M., Fusaro A., Lavazza A. (2023). Asymptomatic infection with clade 2.3.4.4b highly pathogenic avian influenza A(H5N1) in carnivore pets, Italy, April 2023. Eurosurveillance.

[79] Disease Outbreak News. Influenza A(H5N1) in Cats—Poland. https://www.who.int/emergencies/disease-outbreak-news/item/2023-DON476#:~:text=Some%20cats%20developed%20severe%20symptoms,both%20neurological%20and%20respiratory%20signs.

[80] Neumann G., Kawaoka Y. (2015). Transmission of influenza A viruses. Virology.

[81] HAIRS Risk Statement: Avian Influenza A(H5N1) in Livestock. https://www.gov.uk/government/publications/hairs-risk-statement-avian-influenza-ah5n1-in-livestock/hairs-risk-statement-avian-influenza-ah5n1-in-livestock.

[82] Plaza P.I., Gamarra-Toledo V., Euguí J.R., Lambertucci S.A. (2024). Recent Changes in Patterns of Mammal Infection with Highly Pathogenic Avian Influenza A(H5N1) Virus Worldwide. Emerg. Infect. Dis..

[83] Imai M., Watanabe T., Hatta M., Das S.C., Ozawa M., Shinya K., Zhong G., Hanson A., Katsura H., Watanabe S. (2012). Experimental adaptation of an influenza H5 HA confers respiratory droplet transmission to a reassortant H5 HA/H1N1 virus in ferrets. Nature.

[84] Zhang Y., Zhang Q., Gao Y., He X., Kong H., Jiang Y., Guan Y., Xia X., Shu Y., Kawaoka Y. (2012). Key Molecular Factors in Hemagglutinin and PB2 Contribute to Efficient Transmission of the 2009 H1N1 Pandemic Influenza Virus. J. Virol..

[85] Restori K.H., Septer K.M., Field C.J., Patel D.R., VanInsberghe D., Raghunathan V., Lowen A.C., Sutton T.C. (2024). Risk assessment of a highly pathogenic H5N1 influenza virus from mink. Nat. Commun..

[86] Bussey K.A., Bousse T.L., Desmet E.A., Kim B., Takimoto T. (2010). PB2 Residue 271 Plays a Key Role in Enhanced Polymerase Activity of Influenza A Viruses in Mammalian Host Cells. J. Virol..

[87] Subbarao K., Klimov A., Katz J., Regnery H., Lim W., Hall H., Perdue M., Swayne D., Bender C., Huang J. (1998). Characterization of an Avian Influenza A (H5N1) Virus Isolated from a Child with a Fatal Respiratory Illness. Science.

[88] WHO Cumulative Number of Confirmed Human Cases for Avian Influenza A (H5N1) Reported to WHO, 2003–2024 (Updated 3 May 2024). https://www.who.int/publications/m/item/cumulative-number-of-confirmed-human-cases-for-avian-influenza-a(h5n1)-reported-to-who--2003-2024-3-may-2024.

[89] Potdar V., Brijwal M., Lodha R., Yadav P., Jadhav S., Choudhary M.L., Choudhary A., Vipat V., Gupta N., Deorari A.K. (2022). Identification of Human Case of Avian Influenza A(H5N1) Infection, India. Emerg. Infect. Dis..

[90] Technical Report: Highly Pathogenic Avian Influenza A(H5N1) Viruses. https://www.cdc.gov/flu/avianflu/spotlights/2022-2023/h5n1-technical-report.htm.

[91] Avian Influenza A (H5N1)—Cambodia. https://www.who.int/emergencies/disease-outbreak-news/item/2023-DON445.

[92] Summary of Status of Development and Availability of a(h5n1) Candidate Vaccine Viruses and Potency Testing Reagents. https://cdn.who.int/media/docs/default-source/influenza/cvvs/cvv-zoonotic-northern-hemisphere-2024-2025/h5n1_summary_a_h5n1_cvv_20240223.pdf?sfvrsn=2d559bc4_5&download=true.

[93] Summary of Status of Development and Availability of A(H5) non–A(H5N1) Candidate Vaccine Viruses and Potency Testing Reagents. https://cdn.who.int/media/docs/default-source/influenza/cvvs/cvv-zoonotic---southern-hemisphere-2024/summary_a_h5_cvv_sh24.pdf?sfvrsn=a3dc6046_4&download=true.

[94] Vaccines Licensed for Use in the United States. https://www.fda.gov/vaccines-blood-biologics/vaccines/vaccines-licensed-use-united-states.

[95] https://www.ema.europa.eu/en/search/search/field_ema_web_categories%253Aname_field/Human?search_api_views_fulltext=licensed%20H5%20vaccine.

[96] Influenza Antigen A/Astrakhan/3212/2020 (CBER-RG8A) (H5N8) 23/178. https://nibsc.org/products/brm_product_catalogue/detail_page.aspx?catid=23/178.

[97] Influenza Risk Assessment Tool (IRAT). https://www.cdc.gov/flu/pandemic-resources/national-strategy/risk-assessment.html.

[98] Saville M., Cramer J.P., Downham M., Hacker A., Lurie N., Van der Veken L., Whelan M., Hatchett R. (2022). Delivering Pandemic Vaccines in 100 Days—What Will It Take?. N. Engl. J. Med..

[99] CEPI-Delivery Pandemics Vaccines in 100 Days. https://cepi.net/wp-content/uploads/2022/11/CEPI-100-Days-Report-Digital-Version_29-11-22.pdf?swcfpc=1.

